# Supplementary material for: ATO Increases ROS Production and Apoptosis of Cells by Enhancing Calpain-Mediated Degradation of the Cancer Survival Protein TG2
Source: Int J Mol Sci. 2023 Jun 30;24(13):10938. doi: 10.3390/ijms241310938 (PMC10341930; doi:10.3390/ijms241310938)
Supplement: Supplementary file 1 [file ijms-24-10938-s001.zip › ijms-2363988-supplementary.pdf]

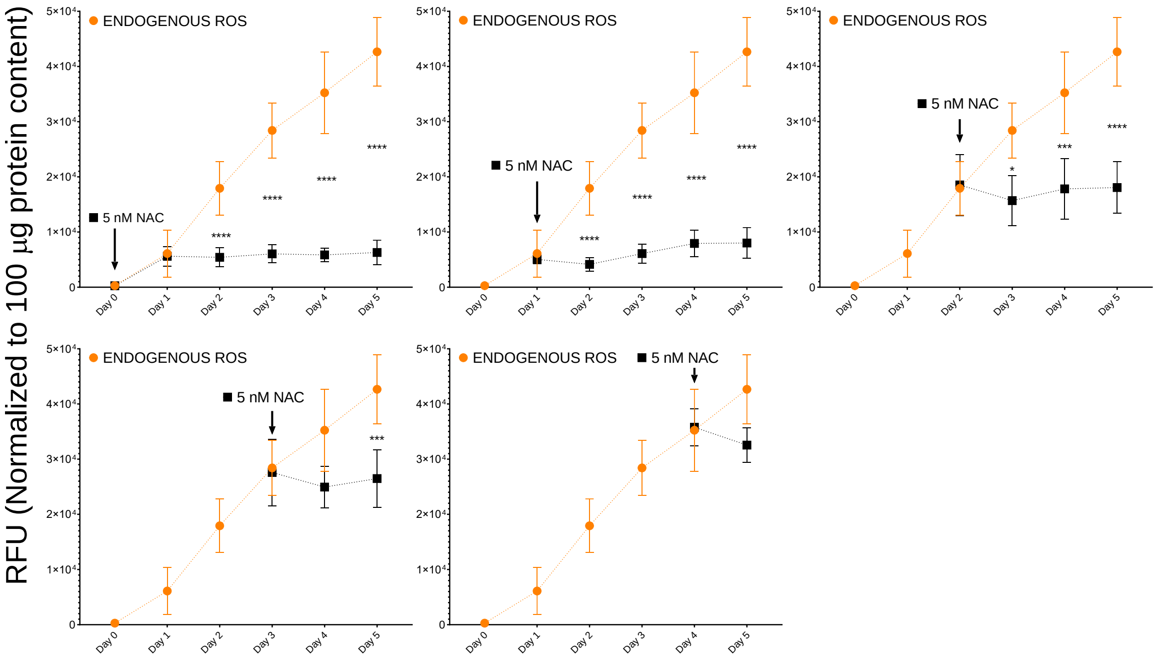

Supplementary Figure S1. Endogenous ROS production is inhibited by 5 nM NAC treatment. *NB4 WT cells were treated with ATRA + ATO 2.0 for five days. The production of endogenous ROS was determined for each cell line using a DCFDA-based method and reported in relative fluorescence units (RFU). The graphs are representations of mean ± SD values normalized to 100 µg protein of total cell lysate content. NB4 TG2 WT cells were treated with ATRA 1 µM+ATO 2.0 µM and ATRA 1 µM+ATO 2.0 µM + 5 nM NAC for five days. Endogenous ROS values were measured on each day in triplicate (n=5). Statistical significance was determined via two-way analysis of variance (ANOVA; Bonferroni post-hoc test; ATRA+ATO vs. ATRA + ATO + NAC * p < 0.05, ** p< 0.01 and *** p < 0.001, **** p < 0.0001).*

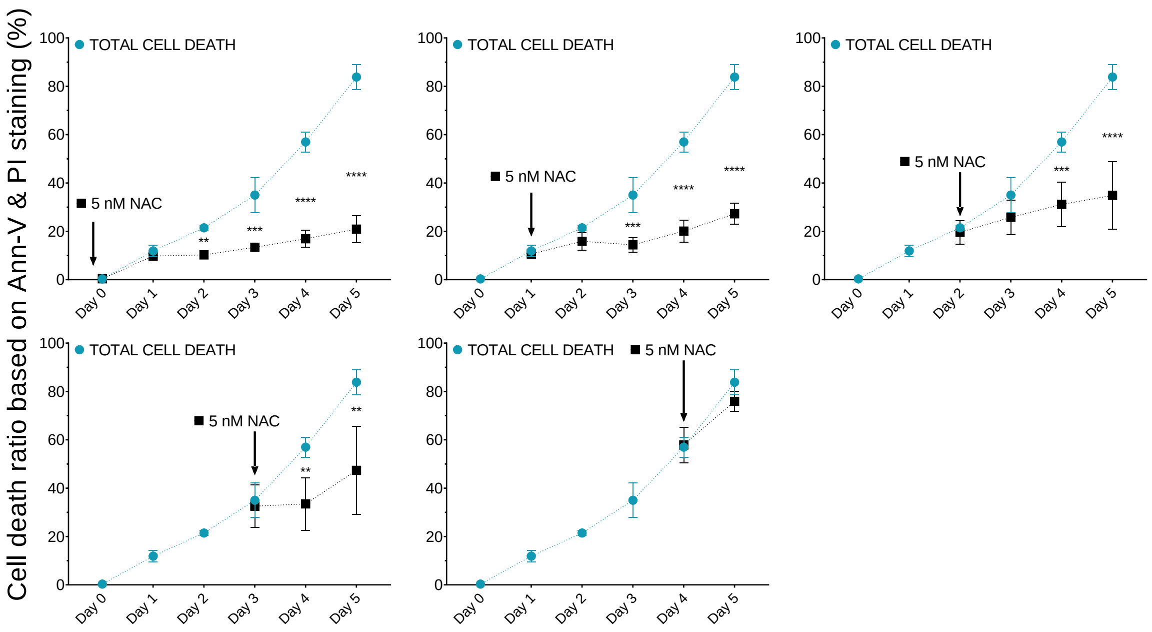

Supplementary Figure S2. Oxidative stress induced Cell death can be attenuated by the administration of 5 nM NAC. *NB4 WT cells were treated with ATRA + ATO 2.0 for five days, harvested, and labeled with FITC/PE-conjugated Annexin-V/PI for 15 minutes. Cells were analyzed on a BD FACSCalibur™. Apoptotic features were evaluated by the size and granularity of the NB4 cells, followed by gating out the FITC/PI-positive cell population. Dead cell ratios were measured each day in triplicate (n=5). Statistical significance was determined via two-way analysis of variance (ANOVA; Bonferroni post-hoc test; ATRA+ATO vs. ATRA + ATO + NAC * p < 0.05, ** p< 0.01 and *** p < 0.001, **** p < 0.0001).*

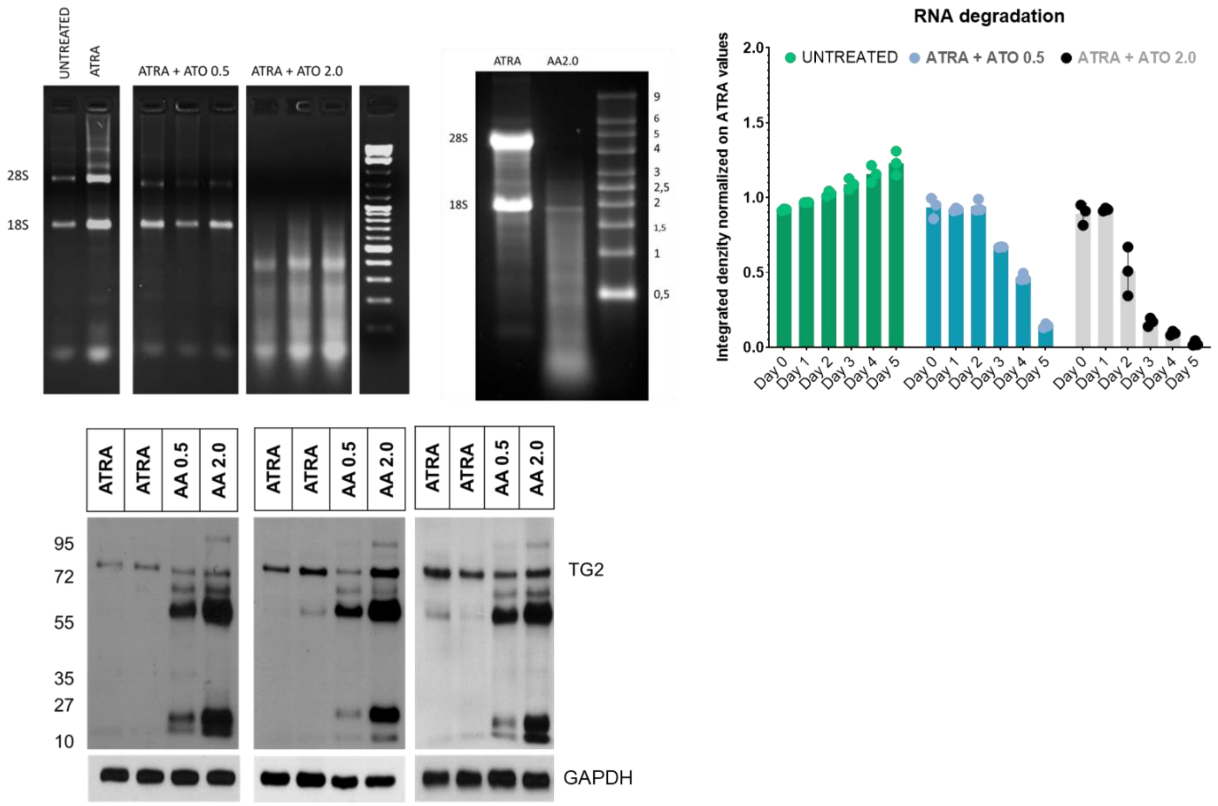

***Supplementary Figure S3. TG2 degradation upon ATO-induced oxidative stress and calpain activation** (left upper panel). Total RNA was isolated by the TRIZOL method from untreated (undifferentiated), ATRA, ATRA+ ATO 0.5 µM and ATRA + ATO 2.0 µM treated NB4 WT cells. Samples were fixed with form-amine solution and electrophoresed on 2% agarose gel. Results show the S28 and S18 ribosomal parts from DAY3 samples, which are degraded upon high concentrations of arsenic-induced oxidative stress. (right upper panel) Densitometry shows the changes upon combined treatment and the disappearance of the intact components from the samples, proving degradation due to arsenic treatment. The values were analyzed by Image J software version 2.4.5 and calculated as a relative integrated optical density. ATRA values were used to normalize the values, considering the S28 and S18 parts. (Left-bottom panel). WB results below show three biologicals in parallel upon ATRA, ATRA + ATO treatments. The blots represent the day 5 samples, where a monoclonal anti-TG2 antibody was used with 20 µg proteins in each well.*
